# Ethanolic Extract of Xylopia aethiopica Attenuated Aluminum-Induced Ovarian Toxicity in Adult Female Wistar Rats

**DOI:** 10.5935/1518-0557.20240024

**Published:** 2024

**Authors:** Leko Bankole Japhet, Gideon Olamilekan Oluwatunase, Tejumade Olubusayo Adejayan

**Affiliations:** 1 University of Medical Sciences Ondo, Ondo, Nigeria

**Keywords:** ovarian toxicity, Xylopia aethiopica, aluminum chloride, zinc sulphate

## Abstract

**Objective:**

Aluminum is a widely used metal in homes and industries. *Xylopia* aethiopica is an important medicinal plant with antioxidant properties. The objective of this study is to investigate the ameliorative potential of *Xylopia* aethiopica on aluminum-induced ovarian toxicity in Wistar rat.

**Methods:**

Twenty-five rats were randomized into five groups with five rats per group. Group 1 received only distilled water; Group 2: received 150mg/kg of aluminum chloride; Group 3: received 150mg/kg aluminum chloride with 100/kg *Xylopia* aethiopica seed extracts; Group 4: received 150mg/kg aluminum chloride with 50 mg/kg *Xylopia* aethiopica seed extracts, and Group 5: received 150mg/kg aluminum chloride with 50mg/Kg zinc sulphate. For twenty-one days, all administrations were done orally. The rats were then sacrificed following chloroform anesthesia. The ovaries were harvested for histological examination.

**Results:**

The data were analyzed on IBM SPSS software version 21 and the differences in mean values were considered significant at *p*<0.05. *Xylopia* aethiopica extracts significantly (*p*<0.05) reversed the detrimental effects of aluminum chloride on luteinizing hormone, follicle stimulating hormone, progesterone and estradiol. The histological analysis of the ovaries showed a significant improvement in rats treated with *Xylopia* aethiopica extract and zinc sulphate. However, *Xylopia* aethiopica was more effective in a dose-dependent manner.

**Conclusions:**

This study suggests that *Xylopia* aethiopica has ameliorative potential on aluminum-induced toxicity in the ovaries of adult female Wistar Rats.

## INTRODUCTION

Aluminum (Al) is the third most common chemical element on Earth after oxygen and silicon and the most widely used nonferrous metal. It is said to be the most abundant metal in the environment and is frequently accessible to animal and human populations (Maya *et al*., 2016). It is commonly used due to its light density and coagulating property. Al is naturally available through the erosion of Earth crust. In addition, Al can be found in the air due to human and industrial activities such as mining, agriculture, coal combustion, iron and steel foundries, brass and bronze refineries, motor vehicle emissions, and the melting, filing and sawing of aluminum metals (Botté *et al*., 2022). Al compounds are also widely used as coagulating agents in the treatment of water for drinking, food additives, and is found on foods naturally containing Al. The use of aluminum cookware, utensils and wrappings could also contribute to aluminum contamination (Cech & Montera, 2000). Despite its multi-organ toxic effects, it has its largest impact on the reproductive system, where it causes hormonal imbalance which can lead to infertility.

Anatomically, the ovary is the female gonad. It is a paired intraperitoneal endocrine organ typically found in the left and right lower quadrants of the abdomen, respectively. The ovaries play a fundamental role in reproduction and in the production of hormones including estrogen, testosterone, inhibin, and progesterone (Moore *et al*., 2014).

Aluminum accumulates in endocrine glands and causes damage to the glands through oxidative stress, thereby decreasing the level of the hormones secreted into the bloodstream for action at the target organs, causing organ failure (European Food Safety Authority, 2011). There are reports of testicular and ovarian failure from inadequate androgenic hormone levels and decreased androgen receptor function (Boran *et al*., 2013). Fu *et al*. (2014), in which aluminum exposure damaged the ovarian structure, disrupted the metabolism of iron, zinc and copper in the ovary, and decreased ovarian ATPase activity and the expression of androgenic receptors for FSH and LH. All such events might lead to infertility due to the inhibition of ovulation and corpus luteum development. On the whole, aluminum toxicity causes lesions in the ovaries resulting in impairment of ovarian function related to ovulation, with the consequence of reproductive inefficiency associated with failed pregnancy and poor fetal development both during oocyte development and post-fertilization. Furthermore, the fetal contribution to the placenta, fetal limb growth, and neural tube development are hindered in females challenged with zinc deficiency during pregnancy (Chen *et al*., 2010).

*Xylopia aethiopica* is an aromatic tree which grows up to 15-30 m high and about 60-70 cm in diameter. It is native to the lowland rainforest and moist fringe forest in the savanna zones of Africa, although it is largely found in Central and Southern Africa (Okwari *et al*., 2013). Its common names include African pepper, Guinea pepper, spice tree, Negro pepper, African pepper and Senegal pepper (Jirovetz *et al*., 1997). Preliminary screening of the phytochemical constituents of the fruits of *Xylopia aethiopica* showed the presence of cardiac glycoside, flavonoids, phlobatannins, tannins, phenol, anthraquinones, saponin and steroids, but an absence of terpenoids and alkaloids. It has also been reported that these compounds are mostly secondary metabolites, which are capable of producing definite physiological actions on the body and are the most important bioactive constituents of natural products (Koba *et al*., 2008; Ekpo *et al*., 2012). The presence of these metabolites suggests great potential for use in phytomedicine.

The fresh and dried fruits, leaf, stem bark and root bark essential oils in *Xylopia aethiopica* produce various degrees of antimicrobial activities. Similarly, anti-anaphylactic and anti-inflammatory effects from the aqueous ethanol extract of the fruit of *Xylopia aethiopica* (Annonaceae) in mice has been documented (Ekeanyanwu & Etinajirhevwe, 2012). Findings suggested that *Xylopia aethiopica* inhibits mast cell-dependent immediate allergic reactions and exhibit anti-inflammatory effects through the inhibition of histamine release from mast cells via the stabilization of the cell membrane (Obiri & Osafo, 2013). In this study, we investigated the comparative ameliorative potential of *Xylopia aethiopica* and zinc on rats with aluminum-induced ovarian toxicity.

## MATERIALS AND METHODS

### Experimental Animals

Twenty-five (25) healthy female Wistar rats (weighing 120-150g) were obtained from the animal house at the University of Medical Sciences, Ondo. The animals were kept in a wired cage at the same facility for a week to acclimatize before the start of the experiment. The animals were housed under standard laboratory conditions, on dark and light cycles of 12 hours, fed rat pellets and provided water *ad libitum*.

### Plant collection and preparation

The dried fruits of *Xylopia aethiopica* were purchased at Iya Laje Central Market, Ondo state, Nigeria. They were authenticated in the Department of Biological Science, Faculty of Sciences, University of Medical Sciences Ondo State and a voucher specimen number (UNIMED PB TH No: 008) was allocated to the plant specimen. The dried fruits were carefully de-seeded and pods were discarded. The seeds were pounded into small pieces using a wooden mortar and pestle and ground into a coarse powder using a mechanical grinding machine.

### Extraction of Plant Materials

Extraction was performed using 70% ethanol. 200g of the coarse powdered seed was weighed out to be 129.5g and extracted in 130ml of ethanol (W/V) via maceration for 72 hours in an air tight container. The mixture was filtered with Whatmann No 3 filter paper. Using a water bath, the ethanol filtrate was concentrated at a low temperature of 45^o^C under reduced pressure, which yielded 6g of a jelly-like extract using the formula:

Percentage yield = mass of extract (g) /mass of powdered sample multiplied by 100

### Preparation of the Aluminum Chloride Solution

The aluminum chloride solution was prepared by dissolving ten gram (1g) of aluminum chloride in 100ml of distilled water. The aluminum chloride solution was administered at a dosage of 150mg/kg of rat body weight.

### Chemical

Aluminum chloride was obtained from Pascal Scientific Limited, opposite Akure south local government, Akure.

### Experimental Design

Twenty-five (25) adult female Wistar rats (weighing 120-150g) were categorized into five groups with five rats in each group. Group 1 (control group) received distilled water and rat pellets only. Group 2 received 150mg/kg of body weight of aluminum chloride daily for two weeks orally. Group 3 received 100 mg/kg of body weight of *Xylopia aethiopica* simultaneously with 150mg/kg of body weight of aluminum chloride orally. Group 4 received 50mg/kg of body weight of *Xylopia aethiopica* simultaneously with 150mg/kg of body weight of aluminum chloride orally. Group 5 received 50mg/kg of body weight of zinc sulphate simultaneously with 150mg/kg of body weight of aluminum chloride orally.

### Animal Sacrifice and Blood Collection

Twenty-four hours after the end of the experiment, the animals were weighed on an electrical sensitive weighing scale. Each rat was then anesthetized with Diethyl-ether and sacrificed. Blood samples were collected from the animals using sterile syringes and needles by cardiac puncture through the mid-clavicular line into plain sample bottles, which were left in an upright position for 120 minutes at room temperature to clot. The clotted blood was thereafter centrifuged at 2000 rpm for 10 minutes using a bench top Uniscope Laboratory centrifuge (Model 802, Surgifried Medicaid and Essex, England). The serum obtained from the respective samples was carefully removed using Pasteur pipettes and placed into their respective labeled plastic specimen bottles and stored in a bio-freezer until analysis.

### Organ Harvesting and Tissue Processing

Using a midline abdominal incision, the abdominal cavity was opened to expose the two ovaries, which were excised and fixed in 4% buffered paraformaldehyde, dehydrated in various grades of ethanol, cleared in benzene, infiltrated and embedded in paraffin wax. The tissue blocks were mounted on wooden blocks and trimmed to size at 20µ thick. They were sectioned on a rotatory microtome at 7µ thick. The sections were stained with Hematoxylin and Eosin. Photomicrographs were taken using a 5 mega pixel Amscope digital scope mounted on an Olympus microscope.

### Statistical Analysis

The analysis was done on IBM SPSS software version 21. Both descriptive and inferential statistics (ANOVA) were done on all obtained data. Turkey’s multiple comparison was used to test for statistically significant differences between control and experimental groups. The data were represented as Standard Error of Mean and the level of differences were considered significant at *p*<0.05.

## RESULTS

### Effects of *Xylopia aethiopica* treatment on the hormonal activities of Wistar rats with aluminum-induced ovarian toxicity

**Figure 1:**
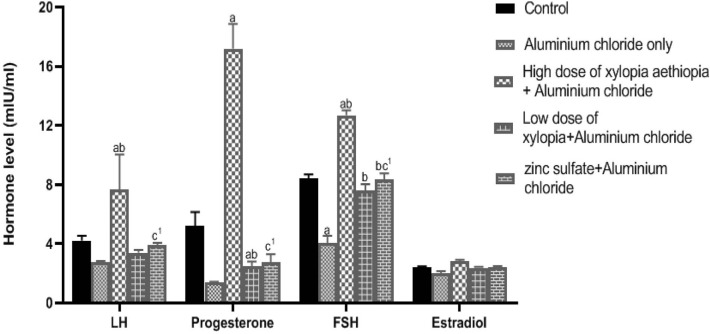
Effect of treatment with seed extract of Xylopia aethiopica on hormonal activity in female Wistar rats with aluminum chloride-induced ovarian toxicity.

Figure 1 shows that the serum LH, progesterone, FSH, and estradiol levels in the group treated with aluminum chloride decreased. However, only the reduction of serum FSH was significant (*p*<0.05) when compared to controls. On the contrary, treatment with Xylopia aethiopica extract caused a significant increase (*p*<0.05) in serum LH, progesterone and FSH levels when compared to controls and the rats given zinc sulphate.

a=Other groups vs. controls (*p*<0.05)

b=Other groups vs. AlCl3 group (*p*<0.05)

C^1^ = ZnSO4+ AlCl3 vs. High dose XA + AlCl3 group (*p*<0.05)

All other comparisons did not yield statistically significant differences.

### Effects of *Xylopia aethiopica* treatment on the histology of Wistar rats with aluminum-induced ovarian toxicity

**Figure 2:**
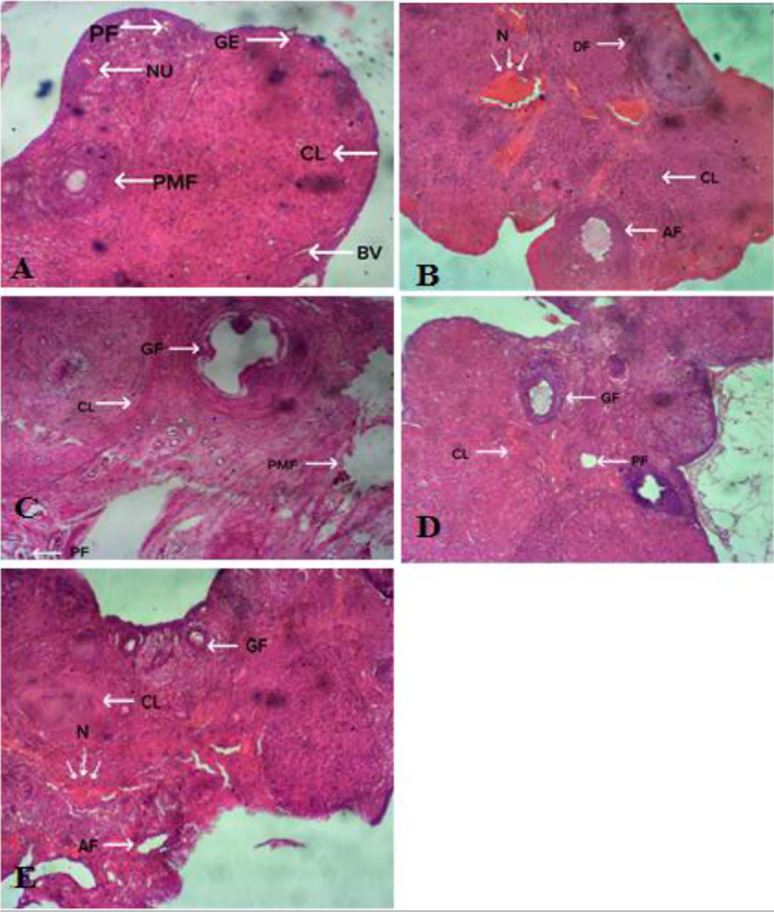
A-E. Photomicrographs of the ovaries of Wistar rats. A. Section of ovaries of rats in Group 1 (Control) showing normal ovarian histology. PF: Primary Follicle; PMF: Primordial Follicle; BV: Blood Vessel; NU: Nucleus; GE: Granulosa Epithelium; CL: Corpus Luteum. B. Section of ovaries of rats in Group 2 (aluminum chloride only) showing N: area of necrosis; CL: Atretic Corpus Luteum; AF: Follicle; DF: Degenerating Follicle. C. Section of ovaries of rats in Group 3 (aluminum chloride + 100mg/kg *Xylopia* aethiopica) PMF: Primordial follicle; GF: Germinating Follicle. PF: Primary Follicle; CL: Corpus Luteum. Features show a regeneration of follicles and other cells and increased number of Primary Follicles (PF). D. Section of ovaries of rats in Group 4 (aluminum chloride + 50mg/kg *Xylopia* aethiopica) showing PF: Primary follicle; GF: Germinating Follicle; CL: Corpus Luteum. Features show mild areas of degeneration in the cortex within the follicles. E. Section of ovaries of rats in Group 5 (aluminum chloride + 50mg/kg zinc chloride). AF: Atretic Follicles; GF: Germinating Follicle; CL: Corpus Luteum; N: Necrosis. Features show Antral Follicle (AF) and area of Necrosis. Growing Follicles (GF) are visible.

The photomicrograph in Figure 2A shows a control rat with normal ovarian architecture, ovarian tissue with a clear primary follicle (PF), primordial follicle (PF), corpus luteum (CL) and blood vessels suggestive of normal ovarian function. Figure 2B shows a section of an ovary from a rat given aluminum chloride, with widespread degeneration and necrosis of follicular cells and atresia of the corpus luteum, suggestive of poor ovarian function. Images from the rats treated with *Xylopia aethiopica* (Figures 2C and 2D) show regeneration of follicular cells and other cell components and increased primary follicle growth. The increase was dose-dependent, with the group receiving a higher dose showing greater populations of reviving cellular components. This suggests that *Xylopia aethiopica* improves ovarian function and ameliorates aluminum-induced ovarian toxicity in Wistar rats. Images from the group treated with zinc sulphate (Figure 2E) show corpus luteum atresia, necrosis and mild follicular degeneration with few growing follicles. This might suggest that zinc sulphate does not have a significant ameliorative effect on Wistar rats with aluminum-induced ovarian toxicity.

## DISCUSSION

Physical observation revealed that the animals treated with aluminum chloride were sluggish, less active and with less appetite than the controls. This is an indication of the disruptions seen in the health of these rats. Hormone profiles showed that serum LH, progesterone, FSH, and estradiol levels in Group 2 (rats given aluminum chloride only) were lower than those of controls (Figure 1). However, only the decrease in serum FSH was statistically significant. This is in agreement with the findings from Adeyemo *et al*. (2001), who documented that aluminum chloride disrupts steroidogenesis, inhibits the production of reproductive hormones and defers puberty. Serum LH, progesterone and FSH levels in Group 3 (aluminum chloride and high dose of *Xylopia aethiopica*) increased significantly (*p*<0.05) when compared to those of controls. This agrees with the findings of Okonkwo *et al*. (2021), who reported that higher doses of *Xylopia aethiopica* caused a significant increase in hormone levels. A likely reason for such increase might be the higher levels of phytochemicals such as flavonoids and glycosides in higher doses of the plant extract. Generally, there were decreases in LH, progesterone, FSH levels in Group 4 (aluminum chloride and low dose of *Xylopia aethiopica*) when compared with controls. However, only progesterone levels decreased significantly when compared with controls. This finding is consistent with the work done by Nnodim *et al*. (2013), who reported that the plant extract significantly decreased FSH levels, and the study by Godam *et al*. (2021), who described that *X. aethiopica* extract caused a significant decrease in serum LH and FSH levels and ascribed this effect of the extract to the high content of saponins, which has the ability to inhibit the release of LH. Significant differences were also seen in the hormone profile results between Group 3 (aluminum chloride and high dose of *Xylopia aethiopica*) and Group 5 (aluminum chloride + zinc sulphate), indicating that the plant extract has more substantial curative and therapeutic effects than zinc sulphate.

Histopathology findings of the group given aluminum chloride orally only (Figure 2B) showed an apparent disruption of the histoarchitecture of the ovaries, with marked degeneration and necrosis of follicular cells and highly congested blood vessels throughout the ovaries, with a large number of atretic follicles at different stages of development when compared to the control group. The histological changes in the ovaries of rats administered aluminum chloride is consistent with the findings of Woode *et al*. (2011) and Onyebuagu *et al*. (2014), who studied the effects of sub-chronic aluminum chloride exposure on the ovaries of rats with similar results. Treatment with *Xylopia aethiopica* extract (Figures 2C and 2D) showed regeneration and a noticeable healing process in the histoarchitecture of the follicular cells in a dose-dependent manner, in line with the report of Godam *et al.* (2021). Animals treated with zinc sulphate (Figure 2E) showed a mild improvement, which also confirmed the findings of Omotosho & Olusanya (2022), who reported that zinc supplementation improved reproductive function of female Wistar rats. However, the ovaries of rats treated with *Xylopia aethiopica* had a healthier histoarchitecture than the those of rats treated with zinc sulphate.

## CONCLUSION

This study demonstrated that aluminum chloride caused deleterious changes in the histoarchitecture of the ovaries of rats treated with aluminum chloride. *Xylopia aethiopica* attenuated the impact of aluminum chloride in the group treated with *Xylopia aethiopica* in a dose dependent manner. Zinc had a slight effect with disoriented histoarchitecture. This might suggest that *Xylopia aethiopica* has ameliorative and therapeutic effects on female Wistar rats with aluminum-induced ovarian toxicity.
